# Low-temperature electrodeposition approach leading to robust mesoscopic anatase TiO_2_ films

**DOI:** 10.1038/srep21588

**Published:** 2016-02-25

**Authors:** Snehangshu Patra, Christian Andriamiadamanana, Michal Tulodziecki, Carine Davoisne, Pierre-Louis Taberna, Frédéric Sauvage

**Affiliations:** 1 Laboratoire de Réactivité et Chimie des Solides, Université de Picardie Jules Verne, CNRS UMR 7314, 33, rue Saint Leu, 80039 Amiens, France; 2 Réseau sur le Stockage Electrochimique de l’Energie (RS2E), FR CNRS 3459, France; 3 Université Paul Sabatier, Toulouse III, CIRIMAT, CNRS UMR 5085, 118, Route de Narbonne, 31062 Toulouse cedex 09, France

## Abstract

Anatase TiO_2_, a wide bandgap semiconductor, likely the most worldwide studied inorganic material for many practical applications, offers unequal characteristics for applications in photocatalysis and sun energy conversion. However, the lack of controllable, cost-effective methods for scalable fabrication of homogeneous thin films of anatase TiO_2_ at low temperatures (ie. < 100 °C) renders up-to-date deposition processes unsuited to flexible plastic supports or to smart textile fibres, thus limiting these wearable and easy-to-integrate emerging technologies. Here, we present a very versatile template-free method for producing robust mesoporous films of nanocrystalline anatase TiO_2_ at temperatures of/or below 80 °C. The individual assembly of the mesoscopic particles forming ever-demonstrated high optical quality beads of TiO_2_ affords, with this simple methodology, efficient light capture and confinement into the photo-anode, which in flexible dye-sensitized solar cell technology translates into a remarkable power conversion efficiency of 7.2% under A.M.1.5G conditions.

The seminal work of Honda *et al.*^1^ back to 1972 demonstrated the feasibility to split water molecules by exposing TiO_2_ surface to incident sunlight. This finding has opened up new avenues for integrating this class of material into photonic displays. The significant attentiveness to this material also stems from its 3d^0^ electronic configuration, particularly sensitive to the introduction of punctual defects by doping or by reductive post-thermal treatment. This offers a very unique and easy way for bandgap engineering, providing in theory a solution to its intrinsic drawback of low solar spectrum absorption resulting from its wide indirect bandgap of 3.2 eV^2^. Consequently, this latter can be tuned to a remarkable range either to a narrowed black 1.85 eV bandgap or inversely blue-shifted to 3.8 eV owing to quantum confinement effect taking place below a size threshold of *ca.* 2 nm^3^,^4^,^5^. Since its first integration into dye-sensitized solar cells technology by O’Regan *et al.* in 1991^6^,^7^,^8^, even though different mesostructures have been tailored^9^ and alternate materials scrutinized to date^10^, nanocrystals of anatase TiO_2_ still remain the leading contender affording the highest power conversion efficiency and stability in conjunction with either heteroleptic ruthenium polypyridil complexes or as a scaffold in hybrid organic-inorganic perovskite absorbers^11^,^12^,^13^.

In a typical synthetic procedure, the anatase requires a temperature of a few hundred of degrees in solid-state reactions to get crystallized^14^. This temperature can be lowered by combining temperature with pressure using conventional hydro(solvo)thermal approaches^15^,^16^ or by more controlled sol-gel/thermolysis methods at mild to even room-temperature^17^,^18^. This heating requirement is also true when using electrochemical, vapor or vacuum deposition techniques for preparing functional thin films. It typically undergoes first a deposition of titanium-based clusters, followed by a thermal post-annealing process to crystallize the film^19^,^20^. Consequently, this second step restricts the utilization of a broad range of substrates among the flexible plastics (PEN, PET…)^21^ or natural/synthetics fibres for the smart textiles in which TiO_2_ as a functional material plays a pivotal role. Investigation on TiO_2_ electrodeposition has remained scarce so far owing to the difficulties to manipulate Ti^3+^ in aqueous solution requiring at once highly acidic chemical bath and argon conditions to maintain Ti^3+^ stable against hydrolysis (pH > 2) and oxidation to air^22^. This was partially overcome recently by the use of ionic liquid-based chemical bath including TiCl_4_ as a precursor, even though still requiring the step of post-annealing for getting crystalline films^23^. With the aim to circumvent this critical last step, we recently discussed a mechanism of dehydration of Ti(OH)_4_ particles taking place at room-temperature which leads to well-crystallized nanoparticles of anatase TiO_2_^24^,^25^. Taking advantage of these preceding works, we herein report a very soft electrodeposition procedure, versatile to all kind of conductive substrates, and easy to handle and to scale-up on larger surfaces, which offers highly porous and robust crystalline mesoscopic films of anatase TiO_2_ not only on FTO-conductive glass but also on flexible plastic PEN-ITO (PolyEthylene Naphtalate). Based on optimized electrodeposition conditions, we demonstrate a flexible dye-sensitized solar cells affording a power conversion efficiency of 7.2% under A.M.1.5G conditions when associated to the robust heteroleptic C106 ruthenium dye.

## Results

**Figure 1:**
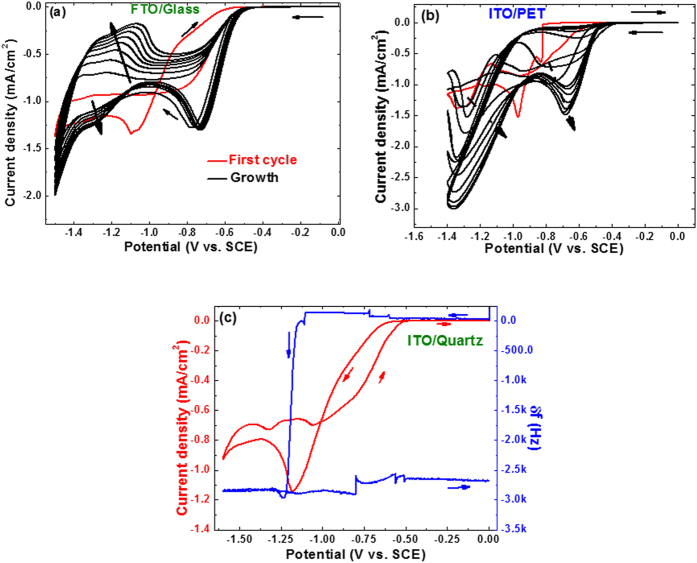
Cyclic voltamperometry experiments on (**a**) FTO/Glass, (**b**) ITO/PET electrode over 10 cycles in a chemical bath of pH = 1.8 consisting of 0.01 mol/L of TiCl_4_ and 0.2 mol/L of KNO_3_ at a sweep rate of 10 mV/s (**c**) ITO coated Electrochemical Quartz Cristal Microbalance (EQCM) measurement of the first cycle.

Figure 1 gathers the successive cyclic voltammograms (CV) recorded between 0 to −1.5 V vs. SCE at ν = 10 mV/s upon a conductive FTO-glass electrode. The aqueous chemical bath is composed of 0.2 mol/L of KNO_3_ and 0.01 mol/L of TiCl_4_. The strategy employed consists on nitrate reduction approach largely scrutinized for ZnO electrodeposition to provide excellent control on the deposit’s texture^26^. Transposed in this work for first time to titanium chemistry, the electrochemical reduction of nitrates NO_3_^−^ to nitrites NO_2_^−^ releases at the electrode’s surface two equivalents of hydroxide responsible for a noticeable local pH increases^26^. In this case, this local pH increases at the electrode/solution interface triggers the precipitation of titanium hydroxide when this latter becomes greater than 3 accordingly to the equations below^27^:


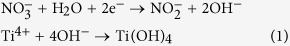


The nitrate reduction is a slow two-electron redox process involving nitrogen-oxygen bond cleavage followed by a structural rearrangement^28^,^29^. This simple approach circumvents the use of both highly acidic and reductive conditions required when using the air-sensitive TiCl_3_ or the pyrophoric derivatives.

Employing the aforementioned chemical bath composition, onset of a cathodic current starts at *ca.*−0.60 V (vs. SCE) on FTO glass electrode or on ITO/PET (Fig. 1). On the first cycle, the cathodic peak shows two components in the current raising, a first attributed to dissolved oxygen reduction followed by nitrate reduction^30^. The maximum peak current reaches a density of *ca.* 1.3 mA/cm^2^ at −1.1 V (vs. SCE). At greater overpotentials, a steady-state regime is systematically noticed before the occurrence of the expected cathodic branch starting at −1.45 V (vs. SCE) corresponding to hydrogen evolution. This intermediate steady-state plateau corresponds to the nitrate mass transport control for which a diffusion coefficient in the range of 10^−5^ cm^2^/s has been reported in the literature^30^,^31^,^32^. One can also notice that the first cycle of the CV features a reduction/oxidation loop which tends to suggest an effective change of the electrode surface. Note that no titanium metal deposit is formed at this range of potentials which could have explained such loop owing to a metal deposition/dissolution process. The first and subsequent cycles are systematically different regardless of the type of electrodes. This is different not only in shape but also in terms of overpotential, corroborating with the modification of electrode surface during the first cycle. The second and ensuing cycles are characterized by a positive shift of the cathodic peak by around 300 mV and a better defined single faradaic phenomenon (Fig. 1a,b). This reveals that when modified, the new surface exhibits greater electrocatalytic activities with respect to nitrate and water reductions. However, the value of steady state current from nitrates mass transport control becomes reduced, thus indicating a lowering in its diffusion flux in the vicinity of the new electrode’s surface. Compared to FTO, ITO/PET electrode shows exactly same trend. On this electrode, the two steps in reduction are better defined with a cathodic peak at −0.83 V corresponding to dissolved oxygen reduction and −0.97 V (vs. SCE) for nitrate reduction (Fig. 1b). A similar shift of +300 mV after the first cycle is also experienced.

To better insight on the redox mechanism during this first cycle, we used electrochemical ITO coated quartz crystal microbalance (EQCM) to monitor the evolution of crystal resonance frequency, therefore the mass by considering Sauerbrey’s equation^33^, as a function of the applied potential at the electrode (Fig. 1c). The first cycle recorded on EQCM reproduces exactly the voltamperogram on FTO, at the exception of a slightly greater overpotential when using the quartz crystal as an electrode. The first shoulder aforementioned, taking place here at *ca.* −0.8 V (vs. SCE), does not affect the quartz resonance frequency. This means that such reduction process is not initiating any deposit upon the electrode which again gives credit to its attribution to oxygen reduction. This falls in contrast to the following faradaic phenomenon which induces a drastic change on the crystal resonance frequency from *ca.* 0 to −3 kHz. This leads to the formation of a clear whitish deposit on the electrode. This film, amorphous by x-ray diffraction, corresponds to Ti(OH)_4_ whose formation is triggered by the local increase in pH contributing to shift the stabilized solvated Ti^4+^ cations towards their hydrolyzed form Ti(OH)_4_ beyond pH = 2^34^.

One advantage of electrodeposition technique over vacuum counterparts is the number of freedom parameters which can offer a very precise control on structural polymorphism, morphology, thickness or porosity of the deposit. In this work, we specifically control the ratio of the concentration of the species involved in electrodeposition, pH of the chemical bath along with cathodic-anodic scan range, various deposition methodologies (galvanostatic, potentiostatic and potentiodynamic) and substrate for obtaining highly porous and well-covering robust deposit.

**Figure 2:**
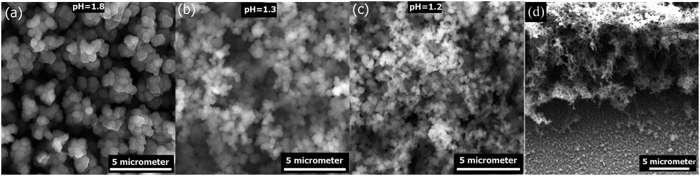
Comparison of SEM micrographs. This compares Ti(OH)_4_ electrodeposited on ITO/PET electrode at a sweep rate of 10 mV/s in the potential window of −0.9 V to 0 V for 40 cycles. The chemical bath is composed of 0.01 mol/L of TiCl_4(aq)_ and 0.2 mol/L of KNO_3_. The pH of the chemical bath was adjusted by adding HCl(aq) to (**a**) 1.7, (**b**) 1.3, (**c**) 1.2. (**d**) is a cross section SEM micrograph of the as-deposited Ti(OH)_4_ film at pH = 1.2.

A first parameter concerns the molar ratio between TiCl_4_ and KNO_3_, adjusted by increasing NO_3_^−^ while keeping Ti^4+^ constant. This ratio controls the proportion between hydroxide produced at electrode surface and the concentration of Ti^4+^ at its surface. Ratios between 1:6 and 1:50 (Ti^4+^ :NO_3_^−^) were investigated. Well covering and robust deposits are not achieved at molar ratio lower than 1:14 and greater than 1:20. The film thickness for the same amount of cycles is increasing with the amount of KNO_3_ as one could expect, eg. 5 μm for 1:6 ratio to 30 μm for 1:20. Keeping an optimized ratio of 1:20, the second parameter, pH of the chemical bath was varied. We found this factor extremely crucial. It is not only changing the film morphology but it also prevents the formation of cracks. A well-controlled pH affords robust, homogeneous and well-covering film. This is illustrated in the Fig. 2 in which we gathered a representative series of SEM micrographs depicting how the film morphology evolves after 40 cycles at different pH from 1.7 to 1.2. Decreasing the pH, although to such a low extent, considerably affects the particle size without modifying the particle morphology. For pH = 1.7, the film is formed of agglomerated well monodisperse spherical particles somehow resembling to small cauliflowers for which the size of the particles is in the range of ~500 nm.

This particle size decreases to ca. 300 nm towards pH = 1.2. This underlines the noticeable influence of the pH on the kinetic competition between nucleation and growth; nucleation being promoted towards more acidic conditions. The cross-section view of this latter film shows a thickness of 15 μm consisting of well-interconnected particles with high porosity all throughout the depth of the film (Fig. 2d). At pH greater than 2, a white precipitate is progressively forming into the chemical bath owing to hydrolysis of Ti^4+^. In opposite, films electrodeposited at pH below 1.2 becomes fragile and crumbly owing to an excessive porosity, likely generated by the promoted hydrogen evolution on the surface of the electrode^35^. This very close relationship between film’s morphology and chemical bath pH also gets retranscribed in the cyclic voltamperograms where more current is systematically observed when the pH is acidified (Fig. S1a). The film thickness increases substantially with the pH from 12.5 to 21 and 25.5 μm thick electrode at pH = 1.2, 1.3 and 1.7, respectively (Fig. S1b). This evolution comes in opposite trend to the evolution of charge density obtained by integration of the voltamperograms over the 40 cycles. This is explained by the competition between nitrate reduction and hydrogen evolution, the latter becoming predominant when moving to low pH. This can be seen also on the electrode surface with the development of hydrogen bubbles forming close to the cathodic to anodic vertex potential. Such a heterogeneous competition between hydrogen formation and nitrate reduction is particularly fascinating as it provides a very new way, without any means of template, to control dense-to-porous and meso-to-macroporosity depending on the potentiostatic conditions, as carefully demonstrated previously on ZnO^35^.

**Figure 3:**
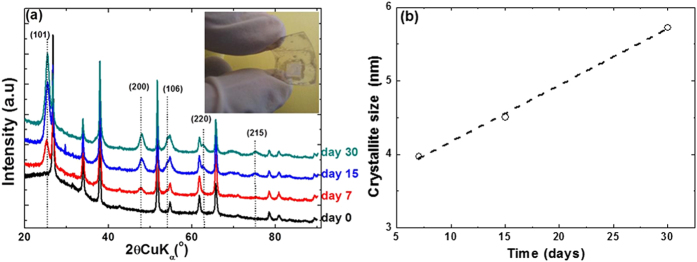
Evolution of the XRD diffractograms (**a**) and crystallite size (**b**) upon ageing of optimized as-deposited films at 80 °C in 0.1 mol/L NH_4_F_(aq)_ solution (substrate FTO glass).

Although the cyclic voltamperometry method is often never considered for electrodeposition since neither the redox process involved is selective owing to potential variations during electrodeposition (by contrast to the potentiostatic method) nor giving a control of the mass transport as for galvanostatic techniques, in this particular case the cyclic voltamperometry revealed to be very suitable to reach covering films with excellent adhesion properties. This is in good agreement with previous experiences by An *et al.*^36^ The value of cathodic to anodic reverse potential is crucial for the deposition quality because of the hydrogen production. This potential is optimum at −1.5 V (vs. SCE) for FTO and −0.9 V (vs. SCE) for ITO/PET in the optimized formulation of the chemical bath. At greater potentials, the deposition yields to dense and poor covering films whereas at excessive low vertex potentials the excessive hydrogen production hampers good film adhesion upon the electrode. The optimized bath composition is perfectly stable for temperatures below 40 °C. Beyond, the titanium tetrachloride is getting gradually hydrolysed. Regardless of the deposition conditions, the as-deposited films were systematically amorphous by x-ray diffraction, as for the other electrodeposition methodology proposed so far. This is not surprising since we recently demonstrated that despite using highly stable ionic liquid chemical bath for which depositions performed up to 150 °C were still insufficient to overcome the energy barrier for electrocrystallization^23^. In this work, we are bypassing this important drawback by means of a simple and versatile method to convert the initial amorphous films to well-crystallized anatase structure by ageing the film into an aqueous solution containing 0.1 mol/L of NH_4_F_(aq)._ This is shown in Fig. 3a in which we collect a series of XRD diffractograms recorded until 30 days of ageing at 80 °C. A gradual crystallization of the anatase polymorph is experienced with a predominance of the most stable and most electro-active (101) diffraction plane^37^. The role of NH_4_F is at once to assist the solid-state dehydration process of the film to onset its crystallization^24^ and at same time to prevent the formation of brookite as part as anatase thanks to fluoride anion capping at the particle surface^24^,^38^,^39^,^40^,^41^,^42^. This solid-state dehydration process, inducing particles crystallization, was further probed by EQCM experiments (Fig. S2). The δf of freshly prepared Ti(OH)_4_/ITO-Quartz was collected in air for 10 minutes until a stable response δf of −12,000 Hz was obtained. Once aged at 80 °C in 0.1 mol/L NH_4_F_(aq)_, the frequency started to increase substantially from −12,000 Hz to −2,535 Hz for 2 hours ageing time and to −1,242 Hz after 7 hours. The increase in δf, i.e. decrease in mass loss (%) by considering Sauerbrey’s equation^33^, is reported as a function of ageing time in Fig. S2b. This evolution describes a single exponential, reaching a plateau after 4 hours of ageing at 80 °C and beyond 7 hours at room-temperature. This suggests that the crystallization process is a two steps process not concomitant with dehydration and that the rate limiting step for crystallization is not dehydration but the subsequent structural rearrangement yielding to the anatase crystal structure.

This means that this method previously developed for anodized titanium foils^42^,^43^ and for powders^18^,^24^ can be perfectly extended to electrodeposited films. All along the duration of ageing, broad diffraction peaks are observed referring to the nano-size nature of the crystallites (Fig. 3). During ageing, the crystallite size increases linearly from 4 to a maximum of 6 nm as described in Fig. 3b. Longer time does not increase further the crystallite size, signifying 30 days is needed to completely convert the pristine amorphous particles to anatase.

**Figure 4:**
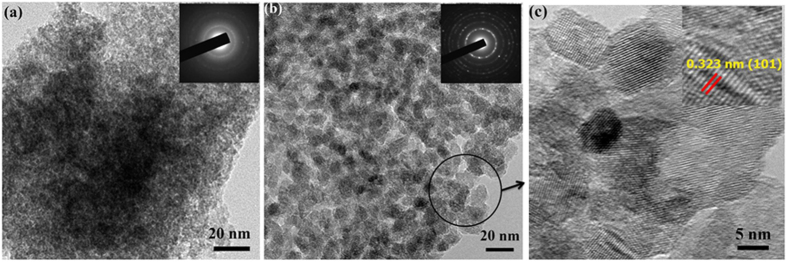
Comparison of TEM micrographs and SAED patterns for (**a**) as-prepared amorphous Ti(OH)_4_ and (**b**) TiO_2_ obtained after 15 days ageing in NH_4_F_(aq)_ (**c**) HRTEM image of TiO_2_ showing dominance of (101) diffraction planes.

The particle morphology does not evolve drastically during this crystallization process by contrast to previous reports on powders showing a solid-state reorganisation of the nanoparticles (Fig. 4)^24^,^44^. In good consistency with XRD, the electron diffraction pattern evolves from diffuse rings, confirming the amorphous character of the as-deposited particles, to well-defined rings ascribed accordingly to the anatase. After ageing, the film is composed of well-crystallized monolithic particles exhibiting rectangular shape of size between 5 to 10 nm. These particles predominantly consist of (101) planes of anatase TiO_2_ (0.323 nm lattice spacing) (Fig. 4c inset). This suggests that the spherical particles of 300 nm size observed by SEM are composed of these aggregated primary particles forming beads. The optical bandgap of the films have been evaluated by UV-visible spectroscopy in diffuse reflectance mode using Kubelka-Munk approach (Fig. S3). The crystallization process reduces the optical bandgap from 3.44 eV of amorphous Ti(OH)_4_^45^ to 3.14 eV in good agreement with the values reported in the literature for anatase^46^,^47^,^48^.

**Figure 5:**
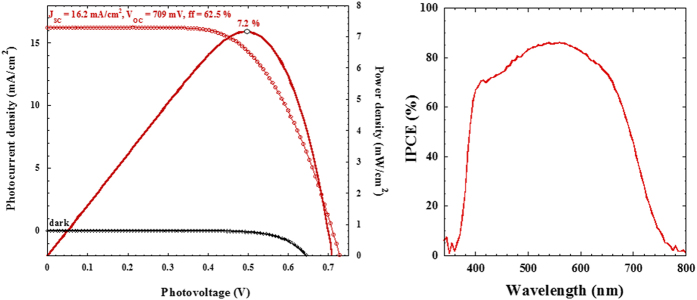
(J–V) curve under dark condition and under A.M. 1.5G illumination of flexible dye-sensitized solar cells composed of electrodeposited TiO_2_ electrode of ca. 12 μm thick sensitized with C106 dye. The related IPCE action spectra is reported.

This two-step method to reach low-temperature crystalline films of mesoscopic anatase TiO_2_ is versatile to all conducting substrates. Taking advantage on the realization of such beads of TiO_2_ for which this kind of morphology has demonstrated outstanding light-to-electricity conversion efficiency and improved charge collection efficiency in dye-sensitized solar cells owing their bi-functionality combining high surface area for high dye-loading and light confinement improvement in the photo-anode accordingly to Mie scattering theory^49^,^50^,^51^,^52^, the performances demonstrated on flexible dye-sensitized solar cells in conjunction with the ruthenium polypyril C106 complex^53^ are among the best values reported so far in the literature on flexible devices^54^,^55^,^56^,^57^ by attaining a power conversion efficiency of 7.2% under standard A.M.1.5G conditions (100 mW/cm^2^) (Fig. 5). The related IPCE action spectra shows a maximum of quantum efficiency of 85% at 556 nm before to decline towards 750 nm. Its integration leads to a value of 15.9 mA/cm^2^, that is excellent agreement with the short-circuit current density measured with 3A class sun simulator. For comparison using same thickness, dye and electrolyte composition, P25-based glass FTO device was achieving 7.1% and 9.3% on 20 nm-based nanocrystalline TiO_2_ without scattering layer (Fig. S4).

## Conclusions

We describe a facile and a versatile two-step methodology using the electrodeposition technique to obtain porous and mesoscopic film of anatase TiO_2_ upon flexible PET/ITO substrate. This method, employing a safer chemical bath, bypasses the current technological obstacle of post-annealing to crystallize the anatase structure. Consequently, this approach paves the way to the utilization of, among others, plastic PEN/ITO substrate or textile fibers, thus of potential high importance for the integration of anatase TiO_2_ into flexible electronics or for smart textiles for instance. We succeeded in obtaining highly desirable porous electrode texture for photonic applications without any means of template, by simply taking advantage of hydrogen evolution to generate mesopores. We also clarify that the crystallization process during thermolysis proceeds in two separated steps, namely dehydration and structural reorganization to form crystalline anatase, the second step being by far the rate limited step. The TiO_2_ produced by this method is composed of meso-macro beads assembly whose properties in photovoltaic dye-sensitized solar cell demonstrated one among the best power conversion efficiencies reported so far on flexible dye solar cells, namely a power conversion efficiency of 7.2% under air mass A.M.1.5G standard conditions (100 mW/cm^2^).

## Additional Information

**How to cite this article**: Patra, S. *et al.* Low-temperature electrodeposition approach leading to robust mesoscopic anatase TiO_2_ films. *Sci. Rep.* **6**, 21588; doi: 10.1038/srep21588 (2016).

## Supplementary Material

Supplementary Information
