# Supplementary material for: Low-temperature electrodeposition approach leading to robust mesoscopic anatase TiO2 films
Source: Sci Rep. 2016 Feb 25;6:21588. doi: 10.1038/srep21588 (PMC4766494; doi:10.1038/srep21588)
Supplement: Supplementary Information [file srep21588-s1.pdf]

Low-temperature electrodeposition approach leading to robust mesoscopic anatase TiO_2 films

Snehangshu Patra,[1,2] Christian Andriamiadamanana,[1,2] Michal Tulodziecki,[1] Carine Davoisne,[1,2] Pierre-Louis Taberna,[2,3] Frédéric Sauvage[1,2]*

[1]Laboratoire de Réactivité et Chimie des Solides, Université de Picardie Jules Verne, CNRS UMR 7314, 33, rue Saint Leu, 80039 Amiens, France

[2]Réseau sur le Stockage Electrochimique de l'Energie (RS2E), FR CNRS 3459, France

[3]Université Paul Sabatier, Toulouse III, CIRIMAT, CNRS UMR 5085, 118, Route de Narbonne, 31062 Toulouse cedex 09, France

*E-mail : frederic.sauvage@u-picardie.fr

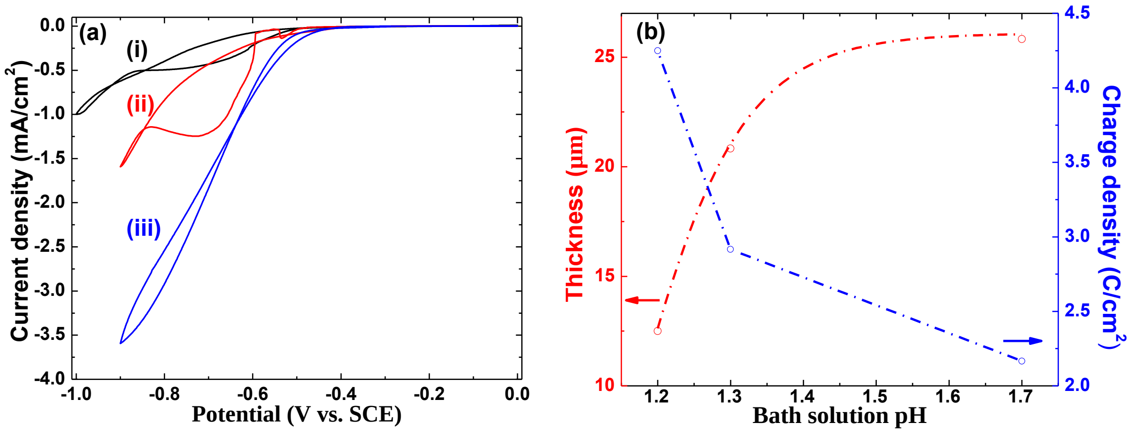

Figure S1. (a) First cycle of the cyclic voltamperogram on ITO/PET electrode in 0.01 mol/L $TiCl_4$ and 0.2 mol/L KNO_3 at various chemical bath's pH with a sweep rate of 10 mV/s. (b) Evolution of film's thickness as a function of chemical bath's pH.

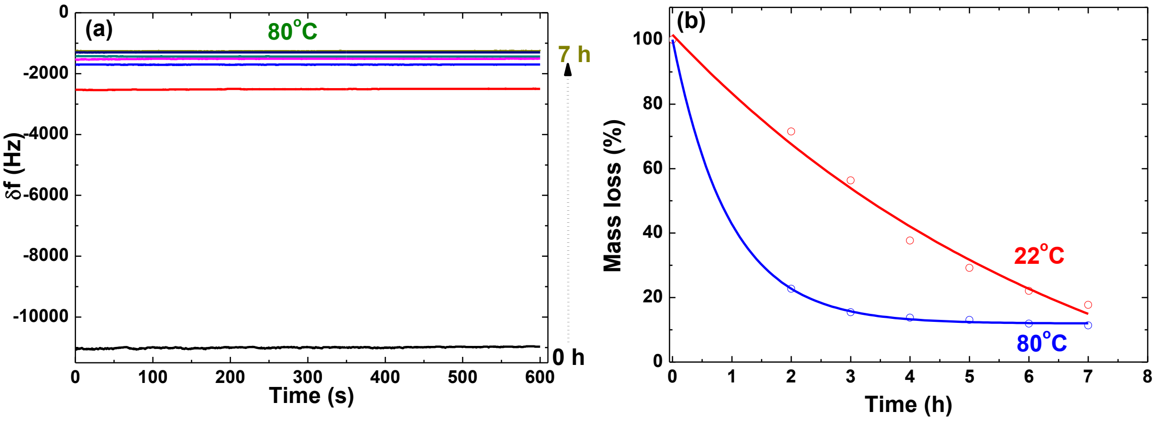

Figure S2. (a) δf and (b) Evolution of mass loss (increase in frequency) vs. time in seconds of as prepared $Ti(OH)_4$-ITO- Quartz crystal electrode during ageing in 0.1mol/L $NH_4F_{(aq)}$ at 80°C. Room-temperature ageing data was also presented in figure b for comparison.

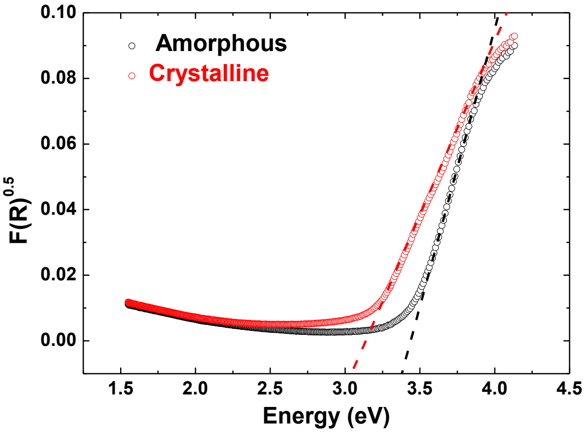

Figure S3: UV-Visible spectra of amorphous $Ti(OH)_4$ and crystalline anatase TiO_2.

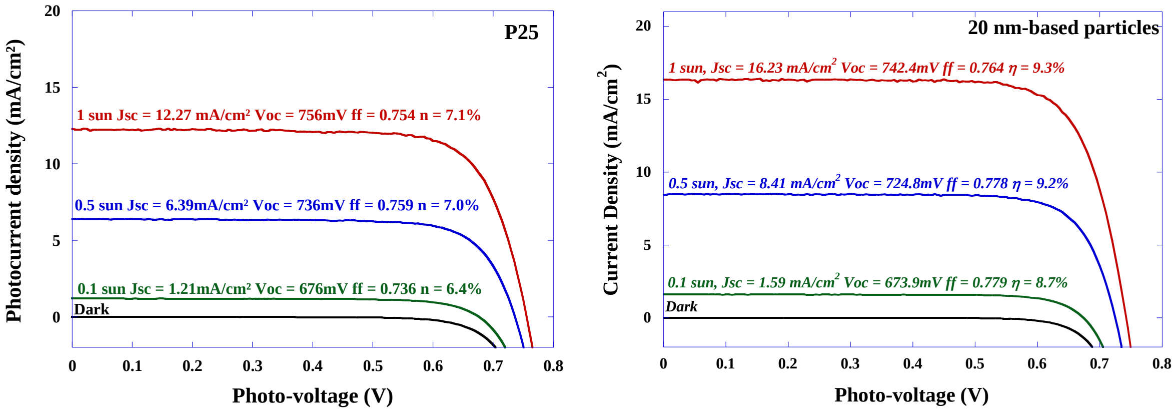

Figure S4: (J-V) curve under different incident light power for a FTO-based glass photo-anode of ca. 10 µm thickness composed of (a) P25 (b) 20 nm-based nanocrystalline anatase TiO_2.

Materials and Methods:

Analytical grade $TiCl_4$ and KNO_3 were purchased from Aldrich and used without further purification. Stock solution of $TiCl_4$ was obtained by adding dropwise 22 mL of pure $TiCl_4$ to 78 mL D.I. water while keeping the container temperature at ~4 °C during the addition. Four different types of electrodes were used as a working electrode (WE): platinum (Pt), stainless steel (SS), fluorine doped tin oxide (FTO) on glass and flexible PET-ITO. The counter electrode (CE) was a platinum gauss ($S \approx 2$ cm^2). The standard saturated calomel electrode (SCE) was used as a reference. Prior to being used, the WE and CE electrodes were cleaned in ethanol in an ultrasonic bath for 1h, and then intensively rinsed with distilled water and ethanol. All the components of the cell were dried in an oven at 60°C for 2 hours prior to any using. The electrochemical measurements were carried out with Biologic VMP3 potentiostat and galvanostat. All the CVs measurements were performed starting from the OCP towards cathodic scan. Optimized TiO_2 films were obtained over 40 cycles at 10 mV/s scan rate using a chemical bath of 0.01 M $TiCl_4$, 0.2 M KNO_3 set at pH = 1.7. The electrodeposited conducting substrate was washed in distilled water and used as it is for ageing. For this, the electrode is typically placed inside a plastic container containing 20 mL 0.01 M $NH_4F_{(aq)}$ and aged inside a temperature controlled oven up to 90°C. The morphology and crystal structure of electrodeposited films were analyzed using a scanning electron microscope FEI Quanta 200 FEG microscope and a Bruker D4 diffractometer using Cu K_a radiation, respectively. The Transmission electron microscopy measurements were performed either on FEI TECNAI F20 S-TWIN FEG microscope or JEOL JEM 2011 LaB_6 microscope operating at 200 kV.

The EQCM measurements were carried out using a commercial SEIKO microbalance with AT-cut 9 MHz quartz covered with ITO on both sides. The electroactive geometric surface

area was equal to S = 0.196 cm^2. A simultaneous measurement of the quartz frequency and current was performed.

UV-Vis absorption spectra were recorded in a diffuse reflectance mode using a Jobin Yvon Cary UV-Vis-NIR spectrometer equipped with an integrating sphere. The photovoltaic properties were evaluated using the C106 ruthenium dye. PET/ITO was purchased from solaronix (ρ =18 Ω/sq) and FTO glass from Nippon Sheet Glass (ρ = 10 Ω/sq). Electrolyte was composed of 1M DMII, 0.03 M I_2, 0.5 M TBP, 0.1 M GuNCS, 0.05 M of LiI in acetonitrile/valeronitrile 85/15 % mixture. The photoanode area is 0.2 cm^2. A black mask was used for measuring (J-V) characteristics with an aperture of 0.25 cm^2. Before overnight sensitization at room-temperature, the photo-anode was dried under vacuum at 80°C during 4 hours. (J-V) curves are measured using Newport Sol3A sun simulator (3A class). The action spectra is measured using a Newport IQE200 spectrometer. For correct measurements, a white light bias of ca. 10 mW/cm^2 was used and the monochromatic light chopped at a 10 Hz frequency. The power conversion efficiency measurements were reproduced over 6 different devices. The values were lying between 6.9 to 7.2 %, the best cell being reported in the main text.
